# Associations between nut intake, cognitive function and non‐alcoholic fatty liver disease (NAFLD) in older adults in the United States: NHANES 2011-14

**DOI:** 10.1186/s12877-021-02239-1

**Published:** 2021-05-17

**Authors:** Sze-Yen Tan, Ekavi N. Georgousopoulou, Barbara R. Cardoso, Robin M. Daly, Elena S. George

**Affiliations:** 1 Institute for Physical Activity and Nutrition (IPAN), School of Exercise and Nutrition Sciences, Deakin University, Victoria 3220 Geelong, Australia; 2 School of Medicine Sydney, University of Notre Dame Australia, New South Wales 2010 Darlinghurst Sydney, Australia; 3 Department of Nutrition, Dietetics and Food, Monash University, 3168 Victoria, Australia

**Keywords:** Nuts, Older adults, Cognition, Non‐alcoholic fatty liver disease, Metabolic‐associated fatty liver disease, Diet quality

## Abstract

**Background:**

Nuts are nutrient-rich and reported to provide some cognitive and cardiometabolic health benefits, but limited studies have focused on older adults. This study investigated the cross-sectional relationship between habitual nut intake, dietary pattern and quality, cognition and non-alcoholic fatty liver disease (NAFLD) in older adults.

**Methods:**

Older adults (≥ 60 years) from the NHANES 2011-12 and 2013-14 cohorts, who had complete data on cognitive function (as CERAD total, delayed recall, animal fluency and digit-symbol substitution test) and variables to calculate the Fatty Liver Index (FLI), an indicator of NAFLD, were included (*n* = 1848). Nut intake and diet quality (Healthy Eating Index 2015) were determined using two 24-hour diet recalls. Participants were categorised into one of four groups based on their habitual nut intake: non-consumers (0 g/d), low intake (0.1–15.0 g/d), moderate intake (15.1–30.0 g/d) or met recommendation (> 30 g/d), with all outcomes compared between these nut intake groups.

**Results:**

Cognitive scores of older adults were the lowest in non-consumers and significantly highest in the moderate intake group, with no further increase in those who consumed nuts more than 30 g/d (*p* < 0.007). FLI was the lowest among older adults with moderate nut intake but the associations disappeared after adjusting for covariates (*p* = 0.329). Moderate nut intake was also associated with better immediate and delayed memory in older adults with high risk of NAFLD (FLI ≥ 60) (B = 1.84 and 1.11, *p* < 0.05 respectively). Higher nutrient intake and better diet quality (*p* < 0.001) were seen with higher nut intake but did not influence energy from saturated fat intake. Factor analysis revealed ‘Nuts and oils’ as one of the four major dietary patterns associated with better cognition and lower FLI scores.

**Conclusions:**

Moderate nut intake (15.1–30.0 g/d) may be sufficient for better cognitive performance, but not NAFLD risk of older adults in the US.

## Introduction

Being physically active [1] and following a healthy diet [2] are two of the most important lifestyle factors to promote healthy aging and enhance quality of life in older adults. However, an official guideline still has not been established to promote physical, mental health and wellbeing in older adults with comorbidities, plus consuming an adequate diet to support good health can be challenging in some older adults [3]. Adding snacks to main meals has been shown to improve the overall dietary intake of older adults [4]. Nuts are nutrient dense (including high amounts of unsaturated fats, fibre, protein, and essential micronutrients) hence improve intake of essential nutrients and contribute to an overall healthier dietary pattern [5–7], and they can be consumed with meals or alone as snacks [8, 9]. This is particularly important in older adults given their increased risk of malnutrition and comorbidities. Indeed, nut intake has been associated with reduced risk of age-related diseases including metabolic disorders, cardiovascular disease risk, cancer risk, and affective and cognitive disorders [10], which may occur through several underlying mechanisms such as reduced inflammation, oxidative stress, and improved cholesterol metabolism, vascular function, and gut microbiome [11–13]. Optimal nutrition and health may contribute to better quality of life in the older adult population.

Regular nut intake has also been linked to lower body weight and body fatness [14]. There is also emerging evidence linking higher nut intake with lower liver fat accumulation including non-alcoholic fatty liver disease (NAFLD) [15–17], but this association remains unknown especially among older adults in the United States. Liver fat accumulation is an important consideration in older adults because NAFLD, which has more recently been proposed as metabolic-associated fatty liver disease [18], has been recognised as an independent risk factor of vascular dysfunction, cardiovascular disease, and cognitive-related disorders [13, 19, 20]. It has been proposed that increased nut consumption improves vascular function including endothelial function, arterial compliance, blood pressure and cerebral vascular function, all of which are risk factors that have been implicated in cognitive impairment and dementia [19]. Indeed, there is epidemiological evidence that links nut intake to better cognitive function in older adults [21–25]. Previous reviews have suggested that this positive association between nuts and cognition may be related to the antioxidant properties of nuts [26, 27]. Furthermore, evidence suggests a relationship between higher liver fat accumulation and poorer overall cognitive function [28], as well as increased risk of cognitive impairment [29, 30]. This highlights the need to examine if fatty liver would modulate the previously-reported positive associations between nut intake and cognitive function of older adults.

The Dietary Guidelines for Americans 2020–2025 recommend nut intake of 5 ounces/week, which is about 30 g/day on most days [31]. This recommended amount is consistent with guidelines in other countries such as Australia [32] and New Zealand [33]. It is worth noting that 30 g also represent a serving of nuts. This level of recommended nut intake is associated with several health benefits such as optimal body weight and metabolic health [34]. However, the association between nut intake in older adults (60 years and over) residing in the US and fatty liver remains unknown. Furthermore, although nut intake has been associated with cognitive function in older adults, previous studies have not examined this association based on the recommended 30 g/day of nuts. Therefore, the primary aim of this study was to examine the independent association between nut intake with cognition and NAFLD in older adults in the United States. Secondary aims were to explore the interrelationship between nut intake, cognition and NAFLD, and to assess nutrient intake and diet quality according to nut intake.

## Methods

### Study participants

This study included cross-sectional data collected from the National Health and Nutrition Examination Surveys (NHANES) conducted in years 2011-12 and 2013-14 by the Centres for Disease Control and the National Centre for Health Statistics (NCHS). The surveys were approved by the NCHS Research Ethics Review Board (Protocol #2011-17), and all participants provided informed consent. NHANES utilises a probability-sampling procedure that provides estimates of health and nutrition status that are representative of non-institutionalised residents in the United States [35]. This study identified a total of 3632 participants aged 60 years and over from both cohorts. Participants were excluded if they had positive serology for hepatitis B, C and D (*n* = 106), alcohol consumption of > 20 g/day for women or > 30 g/day for men (*n* = 217), and only 1-day or unreliable dietary data as defined by NHANES (*n* = 665). Of the remaining 2677 older adults, 829 participants with missing data from at least one or a combination of the following variables were further excluded: hepatitis status (*n* = 825), educational status (*n* = 8), ratio of family income to poverty (*n* = 346), physical activity level (*n* = 2), history of cardiovascular disease (*n* = 22) and type 2 diabetes (*n* = 13), cognitive function (i.e. CERAD Total (*n *= 501), Delayed Recall (*n* = 506), Animal Fluency (*n* = 522), and Digit Symbol Substitution Test (DSST) (*n* = 618), Fatty Liver Index (FLI) (*n* = 689), dietary data on both days or Healthy Eating Index 2015 (*n* = 827), and two-day dietary recall sample weights (*n* = 691). Some participants had missing data for more than one variable listed above. Thus, this study included a final sample of 1848 older adults who met all inclusion criteria and with complete data for analysis.

### Demographics data

Information on racial group (Mexican American, Non-Hispanic White, Non-Hispanic Black, Non-Hispanic Asian, and others), education status (< 11th grade, high school graduate, some college or associates (AA) degree, and college graduate or higher), socioeconomic position indicated as ratio of family income-to-poverty, and household size were collected using demographic questionnaires, which were administered by trained interviewers using a Computer-Assisted Personal Interviewing system.

### Anthropometric measures

Trained health technicians performed weight, height, and waist circumference measurements using standard examination protocols in the Mobile Examination Centre. Height (cm) was measured using a stadiometer, and body weight (kg) using a digital scale. Waist circumference (cm) was measured at the superior lateral border of participants’ iliac crest. During the anthropometric measurements, participants wore a standard examination gown that consisted of a disposable shirt, pants and slippers, with only underwear underneath the gown. Body mass index (BMI) was calculated as the ratio of weight (kg) and height (meters^2^).

### Dietary assessment, nut intake, and diet quality

Dietary intake was assessed using a 24-hour recall method on weekdays and weekend days, administered by trained interviewers using the United States Department of Agriculture’s (USDA) Automated Multiple-Pass Method. Dietary assessment was performed twice for each participant (three to ten days apart), with the first assessment conducted in-person and the second by phone. Dietary recalls were then analysed for energy, macronutrient and micronutrient intake. This study included participants who have reliably completed both dietary recalls for more accurate reflection of nut intake and calculation of diet quality, and dietary intake was reported as the average intake from both 24-hour recalls.

Nut intake, tree and ground nuts (in both whole and butter forms), were estimated from both 24-hour dietary recalls. Nuts included in this study were almonds, almond butter, Brazil nuts, cashews, cashew butter, hazelnuts, macadamias, pecans, pine nuts, pistachios, walnuts, peanuts, and peanut butter. Although each nut type may have slight variations in individual nutrient composition, they are considered to be nutrient-dense and often grouped collectively [3]. To ensure accurate estimation of nut intake from all food sources, this study considered nuts consumed alone, as well as nuts that were used in foods and recipes from the Food Commodity Intake Database (FCID). For example, this method allowed the quantification of almonds included in an almond chicken dish. The average nut intake from both 24-hour diet recall days was calculated, and participants were categorised as nut non-consumers (0 g/day), low (0.1–15.0 g/day) (zero to half a serving), moderate (15.1–30.0 g/day) (half to a serving), or met recommendation (> 30.0 g/day) (more than a serving).

Healthy Eating Index 2015 (HEI-2015), a measure of dietary adherence to the 2015–2020 American Dietary Guidelines, was used to assess the diet quality of participants included in this study. Briefly, HEI-2015 included nine adequacy components (namely ‘total fruit’, ‘whole fruits’, ‘total vegetables’, ‘greens and beans’, ‘whole grains’, ‘dairy’, ‘total protein foods’, ‘seafood and plant proteins’, and ‘fatty acids’) and four moderation components (namely ‘refined grains’, ‘sodium’, ‘added sugars’, and ‘saturated fats’). A maximum of 5 points were awarded to ‘total fruit’, ‘whole fruits’, ‘total vegetables’, ‘greens and beans’, ‘total protein foods’, and ‘seafood and plant proteins’; and a maximum of 10 points for ‘whole grains’, ‘dairy’, ‘fatty acids’, ‘refined grains’, ‘sodium’, ‘added sugars’, and ‘saturated fats’. The total HEI-2015 score ranges from 0 to 100, where higher scores indicate higher consumption of foods from the Adequacy components and lower consumption of Moderation food components [36]. HEI-2015 scores were calculated for both 24-hour dietary recalls and the average scores are presented herein.

### Cognitive function

Cognitive function in older adults (aged 60 years and over) was assessed during NHANES 2011-12 and 2013-14 cycles using four tests: (i) the Consortium to Establish a Registry for Alzheimer’s Disease (CERAD) test, a measure of immediate learning ability, that consisted of three consecutive tests where participants are instructed to read and recall ten words in each test (scores from all three test repetitions were summed and total score ranges from 0 to 30) [37], (ii) the Animal Fluency test, a measure of verbal fluency which is a component of executive function, where participants were asked to name as many animals as possible in one minute [38], (iii) the Digit Symbol Substitution Test (DSST), a measure of processing speed, sustained attention and working memory, in which participants have 2 min to match (pair) symbols to numbers [39], and (iv) the CERAD delayed recall, which provides a measure of delayed memory, where participants were asked to recall the ten words used in the CERAD test after the Animal Fluency and DSST tests were completed (score ranges from 0 to 10) [37].

### Biochemical markers

A kinetic rate method (Beckman Synchron LX20, Beckman UniCel DxC800 Synchron system) was used to measure triglycerides and liver function test markers including alanine aminotransferase (ALT), aspartate aminotransferase (AST), gamma glutamyltransferase (GGT), and total bilirubin, total protein, albumin, and globulin.

### Non‐alcoholic fatty liver disease (NAFLD)

NAFLD describes a condition where excessive fat is accumulated in the liver, and this condition excludes fatty liver due to other causes of liver disease and/or excessive alcohol consumption. Therefore older adults were excluded from FLI calculation if they were tested positive for Hepatitis B, C and D serology, as well as reported alcohol intake more than 20 g/day for females or 30 g/day for males [40]. The risk of NAFLD was predicted using an index validated in epidemiological studies, known as the Fatty Liver Index (FLI) [41, 42]. The FLI is calculated using the following equation [43]:

**Fatty Liver Index (FLI)** = (e ^0.953×LN (triglycerides) + 0.139×BMI + 0.718×LN (GGT) + 0.053×waist circumference − 15.745^) ÷ (1 + e ^0.953×LN (triglycerides) + 0.139×BMI + 0.718×LN (GGT) + 0.053×waist circumference − 15.745^) × 100.

This FLI equation produces a score that ranges from 0 to 100. A FLI < 30 rules out the presence of NAFLD (negative likelihood ratio = 0.2), while a FLI ≥ 60 suggests the likely presence of fatty liver (positive likelihood ratio = 4.3) [43].

### Physical activity

Physical activity was assessed by trained interviewers using the Global Physical Activity Questionnaire that included questions on daily physical activity and sedentary activities. The amount of time (minutes per week) participants spent on moderate- (4.0 METS) or vigorous-intensity (8.0 METS) physical activities was calculated, and categorised as meeting or not meeting the national physical activity recommendations in the United States of 600 METS·min per week (i.e. at least 150 min of moderate-intensity (4.0 METS) or 75 min of vigorous-intensity (8.0 METS) aerobic physical activity per week) [44].

### Smoking status

 Participants’ smoking status was assessed during interview through two questions: ‘Have you smoked at least 100 cigarettes in your entire life?’ and ‘Do you now smoke cigarettes?’. Individuals who responded ‘no’ to the first question were considered as non-smokers; those who answered ‘yes’ to the first but ‘not at all’ to the second questions were considered as ex-smokers; and those who answered ‘yes’ to the first questions and ‘every day’ or ‘some days’ to the second question were considered as current smokers.

### History of cardiovascular disease (CVD) and type 2 diabetes mellitus (T2DM)

 Participants’ history of CVD and T2DM was obtained from an interview. Participants were considered to have a history of CVD if they had been told that they had angina/angina pectoris, coronary heart disease, stroke, congestive heart failure, or heart attack. History of T2DM was based on participants’ self-reported diagnosis of diabetes, or those who did not report T2DM diagnosis but had a fasting HbA1c that was greater than 6.4 % [45].

### Statistical analysis

Data analyses were performed using IBM SPSS 25.0 and STATA 15.0. Categorical variables were presented as frequencies (relative frequencies). For continuous variables, normality was confirmed with a combination of graphical representation and Shapiro-Wilk tests. Continuous variables are presented as means (standard deviation) if normally distributed, or median (1st, 3rd quartile) when normality was not met. Comparisons of categorical variables (racial group, education status, household size, smoking status, physical activity, history of CVD and DM, and FLI categories) between groups were tested using Pearson’s chi-square (or Fisher’s exact test as necessary). To compare the levels of a continuous variable between total NHANES and final study sample, Student’s t-test (or Mann-Whitney U-test when normality not met) was used, while one-way analysis of variance (ANOVA) (or Kruskal-Wallis when normality not met) was used to compare variables nut intake categories. General linear models (ANOVA) were used to compare cognitive function, FLI, nutrient intake and diet quality between nut intake categories. For primary outcomes, i.e. cognitive function and FLI, ANOVA with Bonferroni post-hoc comparison was performed and also controlled for the effects of the following covariates: age, sex, ethnicity, smoking status, physical activity, diet quality (Healthy Eating Index, HEI-2015), BMI (only for cognitive function scores as BMI was included in FLI calculation), education level, household size, ratio of income-to-poverty, and history of CVD and type 2 diabetes. These factors have been selected as covariates because they either have been shown to be associated with the cognitive function and NAFLD, and the adjustment of diet quality is necessary to ensure that the findings can be attributed to nut intake and not because of a healthier diet. To explore whether the relationships between nut intake and cognitive function were moderated by the levels of NAFLD risk, multi-adjusted linear regression models were used. The linear regression models also included the same covariates listed above. Linear regression coefficients were adjusted with probability weights using the 2-day dietary recall weights that were halved due to the combination of two NHANES waves [46]. Nut intake and-sex interaction terms were introduced in the multiple linear models but no effect was found in cognitive function and FLI outcomes, hence data analysis was not stratified by sex. Level of statistical significance was set at alpha = 5 %.

Principal component analysis was performed using the 29 dietary components used in the HEI-2015 calculation. Based on the scree plot, Kaiser-Meyer-Olkin Measure of Sampling Adequacy (0.580) and *p*-value for Bartlett’s test of Sphericity (< 0.001), the data was adequate to perform factor analysis with Principal Components, where the first four major patterns were further explored in this study, which cumulatively explained 25.9 % of the variance. Correlations between dietary patterns and continuous variables (cognitive function scores) were tested using Spearman’s rho, with and without FLI as a random variable.

## Results

Of the 1848 older adults included in this study, 969 were females (52.4 %). Demographic characteristics of the final study sample (*n* = 1848) compared with the NHANES older adult population (*n* = 3632) are summarised in Table 1, and show that there were significant differences in age, racial group, educational status, ratio of family income to poverty, smoking status and proportion meeting physical activity recommendations.

**Table 1:** Characteristics of older adults aged 60 years and over in the NHANES 2011-14 (*n*=3632) and the final population included in this study (*n*=1848)

	Study sample (***n***=1848)	NHANES population (***n***=3632)	P
Age in years, mean (SD)	69.0 (6.7)	70 (7.0)	0.001
Females, n (%)	969 (52.4)	1872 (51.5)	0.532
Racial Group, n (%)			0.001
*Mexican American*	162 (8.8)	336 (9.3)	
*Non-Hispanic White*	943 (51.0)	1648 (45.4)	
*Non-Hispanic Black*	400 (21.6)	871 (24.0)	
*Non-Hispanic Asian*	138 (7.5)	350 (9.6)	
*Other*^*a*^	205 (11.1)	427 (11.8)	
Educational status, n (%)			<0.001
*<11*^*th*^*grade*^*b*^	426 (23.1)	1074 (29.6)	
*High School graduate*	457 (24.7)	836 (23.1)	
*Some college or AA degree*	531 (28.7)	948 (26.2)	
*College graduate or above*	434 (23.5)	766 (21.1)	
Socioeconomic position ^c^	2.2 (1.3, 4.2)	2.0 (0.0, 5.0)	<0.001
Household size, n (%)			0.075
*Lives alone*	461 (24.9)	921 (25.4)	
*2 people in household*	874 (47.3)	1610 (44.3)	
*>2 people in household*	513 (27.8)	1101 (30.3)	
Smoking status, n (%)			0.037
*Never smoked*	954 (51.6)	1815 (50.0)	
*Ex-smoker*	701 (37.9)	1347 (37.1)	
*Current smoker*	193 (10.4)	465 (12.8)	
Meeting PA guidelines, n (%)	668 (36.1)	1198 (33.0)	0.020
History of T2DM, n (%)	521 (28.2)	1023 (28.3)	0.954
History of CVD , n (%)	406 (22.0)	857 (23.7)	0.142

All values are mean with standard deviations (SD) or number (proportions, %) unless stated.

*PA* physical activity, *T2DM* type 2 diabetes, *CVD* cardiovascular disease

^a^Other includes ‘Other Hispanic’ (*n*=191) and other racial groups (*n*=28)

^b^Includes educational status ‘<9^th^ grade’ (*n*=206) and ‘9^th^ – 11^th^ grade’ (*n*=265)

^c^Socioeconomic position indicated as Ratio of Family Income to Poverty, median with first and third quartile

### Nut intake, anthropometric measures, cognitive function, and NAFLD

The median nut intake of older adults in the non-consumers, low, moderate and met recommendation nut intake groups were 0, 3.4, 19.9, and 44.9 g/day respectively. The anthropometric measurements, FLI categories, and liver function test of participants included in this study, according to their nut intake, are presented in Table 2. In terms of NAFLD prevalence, the moderate nut intake group had the highest prevalence of FLI < 30 (rules out NAFLD) and lowest prevalence of FLI ≥ 60 (likely presence of NAFLD). Overall, liver function test values were within the normal reference range.

**Table 2:** Anthropometry, NAFLD risk groups, and liver function test according to nut intake categories

	Total	Nut Intake Categories				P
		Non-consumers (0 g/d)	Low (0.1 – 15.0 g/d)	Moderate (15.1 – 30.0 g/d)	Met recommendation (>30 g/d)	
**Nut intake**						
n (%)	1848 (100%)	814 (44.0%)	669 (36.2%)	182 (9.8%)	183 (9.9%)	-
Median nut intake (g/d) ^a^	7.6 (2.4, 21.4)	0 ^w^	3.4 (0.9, 7.5) ^x^	19.9 (16.7, 23.6) ^y^	44.9 (35.7, 67.3) ^z^	<0.001 ^i^
**Anthropometry**						
Weight (kg)	80.2 (19.3)	80.9 (19.1)	79.2 (19.5)	77.9 (19.1)	82.3 (19.2)	0.056 ^i^
BMI (kg/m^2^)	29.3 (6.2)	29.7 (6.3) ^w^	29.0 (6.3) ^w^	28.5 (6.1) ^w^	28.9 (5.9) ^w^	0.026 ^i^
Waist circumference (cm)	103 (14.6)	104 (14.3) ^w^	102 (14.7) ^w, x^	100 (15.1) ^x^	103 (15.1) ^w, x^	0.015 ^i^
**NAFLD groups**						
FLI<30, n (%)	726 (39.3)	289 (35.5)	278 (41.6)	88 (48.4)	71 (38.8)	0.023 ^j^
30<FLI<60, n (%)	483 (26.1)	220 (27.0)	167 (25.0)	41 (22.5)	55 (30.1)	
FLI>60, n (%)	639 (34.6)	305 (37.5)	224 (33.5)	53 (29.1)	57 (31.1)	
**Liver function test**						
ALT (U/L) ^b^	19.0 (16.0, 25.0)	19.0 (16.0, 24.0) ^w^	19.0 (16.0, 24.0) ^w^	19.0 (15.8, 25.0) ^w^	22.0 (18.0, 27.0) ^x^	0.001 ^i^
AST (U/L) ^c^	23.0 (20.0, 27.0)	23.0 (20.0, 27.0) ^w^	23.0 (20.0, 27.0) ^w^	22.5 (20.0, 26.0) ^w^	24.0 (21.0, 28.0) ^w^	0.023 ^i^
GGT (U/L) ^d^	19.0 (14.0, 27.0)	19.0 (14.0, 27.0) ^w^	18.0 (14.0, 26.0) ^w, x^	16.0 (13.8, 25.0) ^x^	19.0 (15.0, 27.0) ^w, x^	0.043 ^i^
Total protein (g/dL) ^e^	7.03 (0.48)	7.09 (0.49) ^w^	7.00 (0.47) ^x^	6.98 (0.47) ^x^	6.97 (0.49) ^x^	<0.001 ^i^
Albumin (g/dL) ^f^	4.20 (0.29)	4.18 (0.30) ^w^	4.19 (0.29) ^w^	4.23 (0.29) ^w, x^	4.27 (0.27) ^x^	<0.001 ^i^
Globulin (g/dL) ^g^	2.83 (0.47)	2.91 (0.46) ^w^	2.80 (0.47) ^x^	2.75 (0.44) ^x, y^	2.70 (0.47) ^y^	<0.001 ^i^
Total bilirubin (mg/dL) ^h^	0.676 (0.269)	0.672 (0.268)	0.668 (0.259)	0.701 (0.300)	0.696 (0.277)	0.341 ^i^

All values are mean with standard deviations (SD) or number (proportions, %) unless stated. Post-hoc comparisons were performed if overall statistical significance was achieved; values with different superscript letters were significantly different

^a^Median with first and third quartile nut intake of the total population excludes individuals who were non-consumers

^b^ALT – alanine aminotransferase, normal range 7-55 U/L, median with first and third quartile

^c^AST – aspartate aminotransferase, normal range 8-48 U/L, median with first and third quartile

^d^GGT – gamma glutamyltransferase, normal range 8-61 U/L, median with first and third quartile

^e^Total protein, normal range 6.3-7.9 g/dL

^f^Albumin, normal range 3.5-5.0 g/dL

^g^Globulin, normal range 2.0-3.5 g/dL

^h^Total bilirubin, normal value <1.2 mg/dL

^i^Analysis of Variance (ANOVA) tests

^j^Crosstab analysis, chi-square test

**Fig. 1:**
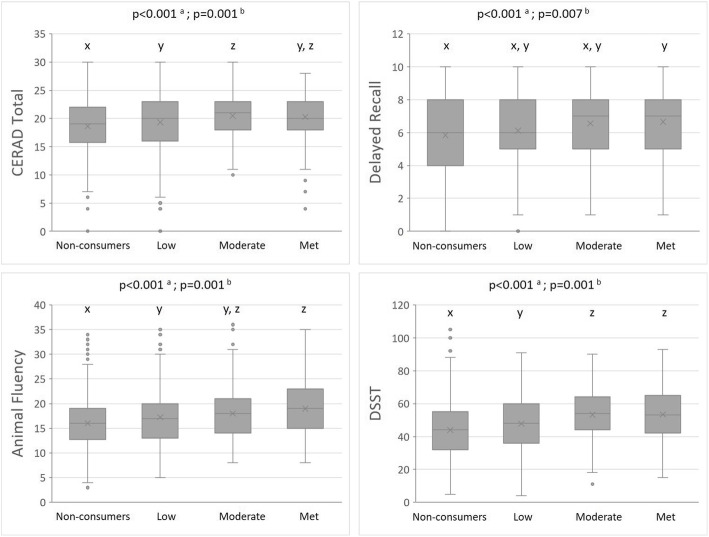
Mean and standard error of CERAD Total, Delayed Recall, Animal Fluency, DSST cognitive test scores in older adults based on nut intake categories: non-consumers (0 g/day), low (0.1–15.0 g/day), moderate (15.1–30.0 g/day), or met recommendation (> 30.0 g/day)

**Fig. 2:**
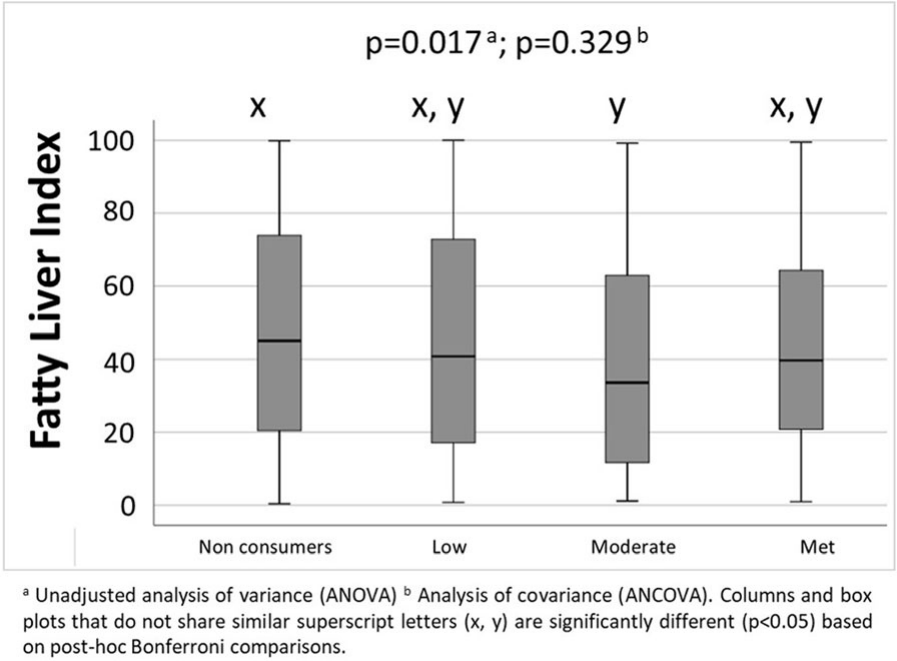
Box plot of fatty liver index in older adults based on nut intake categories: non-consumers (0 g/day), low (0.1–15.0 g/day), moderate (15.1–30.0 g/day), or met recommendation (> 30.0 g/day)

Mean cognitive function test scores based on nut intake groups in older adults are presented in Fig. 1 (all *p* < 0.05 after adjusting for covariates). CERAD total, animal fluency, and DSST scores were significantly higher from non-consumers (lowest scores) to low and moderate (highest scores) intake group, but not the met recommendation group. However, significantly higher score for delayed recall was seen between non-consumers and those who met recommendation. Figure 2 shows the median FLI, which was significantly lower in the moderate nut intake group when compared to non-consumers. However, between-group differences in FLI were not evident after adjusting for covariates.

### Relationship between nut intake and cognitive function based on NAFLD categories

Table 3 shows the associations (beta-coefficient) between cognitive test score and nut intake categories, stratified by NAFLD categories i.e. FLI < 30, 30 ≤ FLI < 60 and FLI ≥ 60. Each variable is independent of other variables included in the linear regression model. The analysis reveals that in older adults who likely had NAFLD (i.e. FLI > 60), moderate nut intake was positively and significantly associated with CERAD Total (beta = 1.84, 95 %CI 0.34–3.34) and Delayed Recall (beta = 1.11, 95 %CI 0.32–1.91) when compared to non-consumers. No other significant associations between nut intake and cognitive function was found in older adults with FLI ≥ 60. Among older adults who did not have NAFLD (FLI < 30), moderate intake and meeting the nut recommendation were associated with better animal fluency scores (beta = 1.98, 95 %CI 0.28–3.70 and beta = 2.04, 95 %CI 0.71–4.00 respectively) than non-consumers. That is, the associations between nut intake and some measures of cognitive function appeared to be moderated by the presence/absence of NAFLD, and moderate nut intake of 15.1–30.0 g/d was linked to better acute and delayed memory when NAFLD was present.

**Table 3:** Associations between nut intake and cognitive function of older adults in the US, stratified by NAFLD risk groups, using linear regression models that also included age, sex, race, Healthy Eating Index scores, history of cardiovascular disease, history of type 2 diabetes, meeting physical activity guidelines, household size, ration of family income-to-poverty, smoking status, and education status

	NAFLD Risk Categories ^a^		
	FLI<30 ^b^ ***n***=726	30**≤**FLI<60 ^b^ ***n=***483	FLI**≥**60 ^b^ ***n=***639
**CERAD Total**			
Nuts consumption group			
*Low vs. non-consumers*	0.10 (-0.89, 1.09)	0.98 (-0.15, 2.10)	-0.73 (-1.81, 0.35)
*Moderate vs. non-consumers*	0.90 (-0.28, 2.08)	0.12 3(-1.85, 2.09)	1.84 (0.34, 3.34)*
*Met vs. non-consumers*	-0.05 (-1.33, 1.23)	1.54 (0.12, 2.96)*	0.69 (-0.77, 2.15)
**Delayed Recall**			
Nuts consumption group			
*Low vs. non-consumers*	-0.18 (-0.70, 0.34)	0.25 (-0.50, 1.00)	-0.01 (-0.56, 0.54)
*Moderate vs. non-consumers*	0.25 (-0.43, 0.94)	-1.12 (-2.51, 0.28)	1.11 (0.32, 1.91)*
*Met vs. non-consumers*	-0.18 (-0.95, 0.58)	0.39 (-0.65, 1.44)	0.75 (-0.01, 1.51)
**Animal Fluency**			
Nuts consumption group			
*Low vs. non-consumers*	0.52 (-0.72, 1.75)	1.30 (-0.02, 2.62)	0.09 (-1.46, 1.65)
*Moderate vs. non-consumers*	1.98 (0.28, 3.70)*	0.88 (-1.24, 3.00)	1.54 (-0.27, 3.34)
*Met vs. non-consumers*	2.04 (0.71, 4.00)*	1.75 (-0.41, 3.91)	0.54 (-2.30, 3.39)
**Digit-Symbol Substitution Test**			
Nuts consumption group			
*Low vs. non-consumers*	-0.14 (-3.54, 3.25)	1.96 (-1.65, 5.56)	0.28 (-2.83, 3.39)
*Moderate vs. non-consumers*	3.69 (-0.96, 8.33)	6.94 (2.15, 11.70)*	1.42 (-4.00, 6.84)
*Met vs. non-consumers*	2.63 (-2.18, 7.44)	1.78 (-3.23, 6.80)	1.24 (-3.03, 5.51)

^a^Values are beta-coefficients and 95%CI, and significance indicated as * (*p*<0.05)

^b^Fatty Liver Index (FLI): Rules out NAFLD risk (FLI<30), inconclusive (FLI 30-60), likely presence of NAFLD (FLI>60)

### Nut intake, nutrient intake, dietary patterns, and diet quality

Intake of energy, macronutrients, fat subtypes (saturated, mono- and poly-unsaturated fat), dietary fibre, and alcohol, according to nut intake categories, are reported in Table 4. Dietary cholesterol intake did not differ between nut intake groups (*p* = 0.523). Diet quality based on the HEI-2015 score was the lowest in non-consumers and improved sequentially with increases in nut intake categories indicating higher diet quality. When factor analysis was performed on the dietary intake data from older adults included in this study, four major dietary patterns were identified namely diets that were high in: (1) refined grains, cured meat, cheese, solid fat, and added sugars, (2) legumes (as vegetables and legumes), (3) intact fruit (excluding citrus, melon, and berries), and dark green, red/orange (excluding tomato) and other vegetables, and (4) nuts and oils (excluding solid fat). The adherence to the ‘nut and oils’ dietary pattern was negatively associated with FLI scores (rho=-0.046, *p* = 0.016), and positively associated with CERAD total (rho = 0.146 and r (partial correlation coefficient) = 0.126, both *p* < 0.001), delayed recall (rho = 0.138,and *r* = 0.127, both *p* < 0.001), Animal Fluency (rho = 0.196 and *r* = 0.180, both *p* < 0.001) and DSST (rho = 0.237 and *r* = 0.232, both *p* < 0.001).

**Table 4:** Nutrient intake^†^ and diet quality of older adults according to nut intake categories

	Total	Nut Intake Categories				
		Non-consumers (0 g/d)	Low (0.1 – 15.0 g/d)	Moderate (15.1 – 30.0 g/d)	Met Recommendation (>30 g/d)	P ^‡^
*n (%)*	1848	814 (44.0)	669 (36.2)	182 (9.8)	183 (9.9)	-
Energy (Kcal)	1813 (674)	1633 (636) ^w^	1862 (635) ^x^	1932 (652) ^x^	2314 (697) ^y^	<0.001
Carbohydrate						
*g/day*	225 (86.7)	204 (82.6) ^w^	236 (83.6) ^x^	237 (85.4) ^x^	266 (93.2) ^y^	<0.001
*%E*	50.1 (8.6)	50.4 (9.3) ^w, x^	51.1 (7.9) ^w^	49.2 (7.7) ^x^	45.7 (7.6) ^y^	<0.001
Protein						
*g/day*	72.8 (29.2)	67.4 (28.6) ^w^	73.4 (28.2) ^x^	76.3 (27.7) ^x^	91.3 (28.5) ^y^	<0.001
*%E*	16.4 (4.1)	16.9 (4.4) ^w, z^	16.0 (3.9) ^x^	16.0 (3.5) ^x, y^	16.1 (3.6) ^x, z^	<0.001
Total fat						
*g/day*	70.5 (33.6)	61.7 (31.3) ^w^	70.9 (30.5) ^x^	77.0 (31.6) ^x^	102 (36.2) ^y^	<0.001
*%E*	34.3 (7.4)	33.3 (7.7) ^w^	33.7 (6.7) ^w^	35.7 (6.9) ^x^	39.5 (6.1) ^y^	<0.001
PUFA (g/day)	16.9 (9.1)	14.4 (8.3) ^w^	17.1 (8.5) ^x^	18.5 (7.7) ^x^	25.3 (10.0) ^y^	<0.001
MUFA (g/day)	25.2 (13.0)	21.4 (11.5) ^w^	24.8 (11.4) ^x^	28.2 (11.4) ^y^	40.5 (15.5) ^z^	<0.001
SFA						
*g/day*	22.3 (11.8)	20.3 (11.4) ^w^	22.9 (11.3) ^x^	23.7 (12.5) ^x^	28.0 (12.7) ^y^	<0.001
*%E*	10.8 (3.2)	10.9 (3.4)	10.8 (3.0)	10.8 (3.2)	10.7 (2.9)	0.904
Fibre (g/day)	17.4 (9.2)	15.0 (8.7) ^w^	17.7 (7.9) ^x^	19.2 (9.0) ^x^	25.5 (11.2) ^y^	<0.001
Alcohol (g/d)	10.9 (6.3, 16.8)	10.6 (5.6, 16.8) ^w^	9.73 (6.4,16.2) ^w^	12.8 (7.0,19.3) ^x^	13.0 (7.0, 19.0) ^x^	<0.001
Cholesterol (mg/day)	231 (143, 346)	226 (141, 340)	236 (146, 353)	215 (138, 341)	241 (153, 350)	0.523
Sodium (mg/day)	3073 (1210)	2905 (1250) ^w^	3168 (1174) ^x^	3134 (1153) ^w, x^	3413 (1098) ^x^	<0.001
HEI-2015	54.5 (12.3)	51.1 (11.5) ^w^	54.9 (11.6) ^x^	58.9 (12.1) ^y^	64.1 (12.1) ^z^	<0.001

^†^ Values are mean (standard deviation) for all nutrients, except for alcohol and cholesterol that are reported as median (1^st^ quartile, 3^rd^ quartile). Abbreviations: %E – percent of total energy intake, PUFA – polyunsaturated fat, MUFA – monounsaturated fat, SFA – saturated fat, HEI-2015 – Healthy Eating Index 2015 scores

^‡^ Analysis of Variance (ANOVA) tests. Post-hoc comparisons were performed if overall statistical significance was achieved; values with different superscript letters were significantly different

## Discussion

The primary aim of this study was to investigate the associations between nut intake and cognitive function and NAFLD prevalence in older adults living in the United States. Our analyses revealed that cognitive function scores increased with nut intake up to moderate intake of 15.1–30.0 g/d, and scores did not change significantly beyond this level of nut intake. An exception was delayed recall, where significantly higher score was seen with intake greater than 30 g/d. The positive associations between nut intake and cognitive function in this study of relatively healthy older US adults was consistent with previous observational studies that reported significant associations between nut intake and global cognitive function in older adults with or without mild cognitive impairment [24]. Longitudinal studies also reported that higher nut intake was associated with slower cognitive decline in this population over the study follow-ups [23, 47]. In term of specific domains of cognitive function, positive associations have been reported between nut intake and immediate learning ability [22], as well as processing speed, sustained attention and working memory [22, 48]. A previous study that also included older NHANES data from 1988 to 2002 reported that walnut consumers performed better on reaction time [25].

The results from this study also indicate that moderate nut consumption appears to be associated with lower prevalence of NAFLD measured by FLI in older adults. To-date, although studies that specifically investigated the relationship between nut intake and NAFLD of older adults are very limited, but available evidence from other populations is consistent with our findings, where nut intake is associated with lower NAFLD risks [49, 50]. In addition to cognition and NAFLD, the moderate nut intake group also had the lowest mean weight, BMI and waist circumference, which are established risk factors for these conditions. Therefore, our findings suggest that the same nut recommendation of one daily serving (30 g) on 5 days/week (or about 20 g/d every day in a week) is also suitable to promote better cognition and lower risk of NAFLD in older adults.

However, it should be highlighted that the relationship between nut intake and NAFLD disappeared after adjusting for several potential covariates including older adults’ history of CVD and type 2 diabetes. NAFLD is often associated with these cardiometabolic comorbidities [51], and higher prevalence of NAFLD is often found in individuals with type 2 diabetes and CVD, than those without [52]. This may explain why the associations between nuts and NAFLD disappear after adjusting for history of type 2 diabetes and CVD. This is a novel finding and should be confirmed in future studies. If the association between nut intake and NAFLD is mediated by CVD and type 2 diabetes, these individuals are still likely to benefit from regular nut intake, as previous studies have shown the protective effects of nuts against CVD and type 2 diabetes [13, 53, 54].

NAFLD is a known risk factor for several metabolic and vascular diseases [51], and impaired vascular function is proposed to be a pathway to poorer cognitive function.[19] Indeed, a number of studies have reported poorer cognitive function among individuals with NAFLD [28, 55–57]. In this study, we also examined the inter-relationships between nut intake, NAFLD and cognition. In addition to the positive association between nut intake and cognitive function, we also found early evidence that the associations between nut intake and CERAD total (immediate) and delayed recall were seen in older adults who have FLI ≥ 60 (high NAFLD risk) and consumed moderate amount of nuts when compared to non-consumers. Higher nut intake (met vs. non-consumers) was also associated with better immediate memory (CERAD total) in individuals with moderate NAFLD risk (30 ≤ FLI < 60). This appears to suggest that individuals with higher NAFLD risk may receive the benefits of nuts at a lower level of intake. However, this was not the case in animal fluency and DSST tests. Individuals with negligible NAFLD risk are still likely to benefit from moderate and met level of nut intake on their executive function (animal fluency test), and those with moderate nut intake may enhance the processing speed, sustained attention and working memory of older adults with moderate NAFLD risk. To our knowledge, this is the first study to observe the inter-relationships between these three factors, and it was unclear what may have contributed to the differential benefits of nuts on different domains of cognition, at different level of nut intake, and categories of NAFLD risk. Specifically-designed future studies are needed to confirm our observations so that specific nut recommendations can be made to different groups of older adults based on their NAFLD risk in the future.

We also found differences in nutrient intake across nut intake categories. Overall, we observed higher overall nutrient intake with higher nut consumption in older adults. This is consistent with findings from other studies that nut intake improves overall nutrient intake [3]. Although higher total fat intake was observed with higher nut consumption, percentage energy intake from saturated fat did not differ between nut intake categories, hence not a major concern. Also, higher energy intake in nut consumers did not pose risk for obesity as body weight and BMI were lower with higher nut intake. Again, the negative association between nut intake, body weight and obesity is consistent with other epidemiological studies [58–63], where nut intake (expressed as amount of frequency of intake) was negatively associated with body weight. This may be attributed to increased basal metabolic rate, lower energy/fat absorption, and supressed appetite as a result of nut intake [14]. We also observed improved diet quality with higher nut intake groups in our cohort of older adults, which has been previously reported in studies that included older adults [64] and other populations [65–67]. Using *posteriori* factor analysis, ‘nuts and oils’ was identified as a major, protective dietary pattern. This implies that nuts, as part of an overall diet (indicated by HEI-2015) and as a major dietary pattern, may be the reason to explain the better cognitive function and lower NAFLD risk (FLI scores) in older adults in this study.

Our study has a number of strengths, including categorising nut intake based on the current nut recommendation of about 30 g/day on most days of a week, hence enhancing translation ability of study findings. This study is also one the first studies to examine the potential benefits of nuts on older adults’ cognition and NAFLD, and the inter-relationship between these two health conditions. Few studies have been conducted on nuts and in older adult populations possibly because nuts are generally considered to be unsuitable due to their hard texture and common issues with dentition in older adults [3]. However, our study demonstrates that such a concern was baseless in the general US population given that 56 % of older adults reported consuming nuts in their diet during the NHANES dietary assessment. The higher rate of nut consumption reported in this study may be due to the fact that nut butter (e.g. almond butter, cashew butter and peanut butter) that are texturally suitable for older adults were also included. Furthermore, this is the first study to examine the inter-relationship between nut intake, NAFLD, and cognition in older adults, and hence provides support for future research into these areas. However, this study is not without limitations including that it was observational in nature and thus only associations can be implied and causation cannot be established. It should also be noted that some demographic characteristics of older adults (*n* = 1848) were significantly different (albeit small) in the total older adult samples from both NHANES cycles (*n* = 3632). For the reasons above, the results may not be generalised to the entire US older adult population, and should be interpreted with caution. In NHANES dietary interviews were conducted by trained staff with tools such as portion guide and automated multiple-pass method that ensure the accuracy of recalls. In this study, we included diet recalls that were identified as reliable, and we included two dietary recalls to increase the representativeness of dietary intake. However, nuts are often not consumed regularly and there is a possibility that nuts were or were not consumed on the day before the 24-hour dietary recalls, hence not reflecting habitual intake. This is a common limitation of a 24-hour recall method, but in this study we included diet data from two recalls to minimise this limitation. Finally, like any observational study, there is always a possibility of reverse causation. For example, nuts may be perceived as a high fat food and people who have high BMI may avoid them in an attempt to regulate their body weight, hence explains the relationship between low nut intake and high BMI. However, this is less likely to be a concern for NAFLD as liver fat accumulation is not visible and often unsuspected until a more progressed state.

## Conclusions

Nut intake in line with current recommendations was associated with better cognitive function especially in those identified as having higher NAFLD risk. The potential benefits of nuts in NAFLD is not demonstrated after accounting for CVD and T2DM. This association may at least in part due to improved nutrient intake and diet quality in these individuals with higher nut intake.

## Data Availability

The original NHANES dataset to support this study is available from the National Center for Health Statistics https://wwwn.cdc.gov/nchs/nhanes/default.aspx.

## Abbreviations

ALT: Alanine aminotransferase
ANOVA: Analysis of Variance
AST: Aspartate aminotransferase
BMI: Body mass index
CERAD: Consortium to Establish a Registry for Alzheimer’s Disease
CVD: Cardiovascular disease
DSST: Digit Symbol Substitution Test
FCID: Food Commodity Intake Database
FLI: Fatty liver index
GGT: Gamma glutamyltransferase
HEI-2015: Healthy Eating Index 2015
NAFLD: Non-alcoholic fatty liver disease
NHANES: National Health and Nutrition Examination Surveys
T2DM: Type 2 diabetes mellitus
